# Functional Impairment of Central Memory CD4 T Cells Is a Potential Early Prognostic Marker for Changing Viral Load in SHIV-Infected Rhesus Macaques

**DOI:** 10.1371/journal.pone.0019607

**Published:** 2011-05-13

**Authors:** Hong He, Pramod N. Nehete, Bharti Nehete, Eric Wieder, Guojun Yang, Stephanie Buchl, K. Jagannadha Sastry

**Affiliations:** 1 Department of Immunology, The University of Texas M.D. Anderson Cancer Center, Houston, Texas, United States of America; 2 Department of Veterinary Sciences, The University of Texas M.D. Anderson Cancer Center, Houston, Texas, United States of America; 3 Department of Medicine, University of Miami, Miami, Florida, United States of America; Beijing Institute of Infectious Diseases, China

## Abstract

In HIV infection there is a paucity of literature about the degree of immune dysfunction to potentially correlate and/or predict disease progression relative to CD4^+^ T cells count or viral load. We assessed functional characteristics of memory T cells subsets as potential prognostic markers for changing viral loads and/or disease progression using the SHIV-infected rhesus macaque model. Relative to long-term non-progressors with low/undetectable viral loads, those with chronic plasma viremia, but clinically healthy, exhibited significantly lower numbers and functional impairment of CD4^+^ T cells, but not CD8^+^ T cells, in terms of IL-2 production by central memory subset in response to PMA and ionomycine (PMA+I) stimulation. Highly viremic animals showed impaired cytokine-production by all T cells subsets. These results suggest that functional impairment of CD4^+^ T cells in general, and of central memory subset in particular, may be a potential indicator/predictor of chronic infection with immune dysfunction, which could be assayed relatively easily using non-specific PMA+I stimulation.

## Introduction

Human immunodeficiency virus (HIV) infection exerts profound effects on the immune system in terms of effectively evading the antiviral antibody responses as well as progressively compromising the functional abilities of the cellular immune responses that lead to the development of acquired immunodeficiency syndrome (AIDS) characterized by life-threatening opportunistic infections and malignancies [1], [2]. Even though the mechanism by which HIV replication disables the immune system is still unclear, measurements of the patient's CD4^+^ T cell counts and plasma viral load have long been the benchmarks of immunologic assessment of disease progression and staging of infection [1], [3]. However, reports in the recent past indicated CD4^+^ T cell count and viral load could not fully predict the disease progression, but their functional characterization may be important [4], [5]. Indeed, vigorous HIV-1 specific CD4^+^ T cells responses were shown to be associated with the control of viremia in individuals who received anti-viral treatment during acute HIV-1 infection [6]. Several assays capable to detect antigen-specific T cells have since been developed, and among these flow cytometry based on the ability to detect cytokine production at the single cell level allows simultaneous detection, quantization and phenotypic characterization of CD4^+^ and CD8^+^ T cells [7]. Using flow cytometry to measure intracellular cytokines after stimulation of peripheral blood mononuclear cells (PBMC) with recombinant HIV proteins or overlapping peptide pools, HIV-specific CD4^+^ T cells were detected in individuals with active HIV-1 infection that declined with prolonged viral suppression [8]. These results suggest the importance of functional HIV-1 specific CD4^+^ T cells for the support of anti-HIV-1 effector responses in active disease. However, because HIV-specific T cells are usually in limiting numbers, it is difficult to perform repetitive assays with multiple specific stimulators. Furthermore, it is not yet clear whether antigen stimulation through the T-cell receptor, in combination with co-stimuli, is capable in vitro of inducing adequate cytokine expression measurable by current technologies. Therefore, it is important to identify more prognostic markers based on the use of generalized reagents that reflect changing viral loads and/or disease progression. In this study, using the rhesus macaque nonhuman primate model we investigated whether functional properties of total and memory subsets of CD4^+^ and CD8^+^ T cells would be a potential correlate of viral loads in the blood as a reliable and easier approach. The SIV/SHIV infection of rhesus macaques is the leading animal model of choice by many because it recapitulates many of the features of HIV infection and disease in humans, including CD4^+^ T cells depletion and the slew of opportunistic infections and malignancies [9], [10], [11]. We describe here multicolor flow cytometry analyses combining detailed phenotypic and functional characterization of total as well as memory subsets of CD4^+^ and CD8^+^ T cells from SHIV-infected rhesus macaques with no clinical symptoms but exhibiting markedly differing viral loads associated with differential cytokine production in response to non-specific in vitro stimulation with PMA+I.

## Materials and Methods

### Animals

Rhesus macaques used in this study are colony-bred animals of Indian origin (*Macaca mulatto*) housed in the specific pathogen-free colony fully accredited by the Association for Assessment and Association of Laboratory Animals Care International and the study was conducted according to NIH guidelines on Care and Use of Laboratory Animals. Cryopreserved peripheral blood mononuclear cells (PBMC) samples from a total 20 rhesus macaques experimentally infected with SHIV_89.6P_ or SHIV_KU2_ by the intravenous route in previously published studies [12], [13] were used for the studies in this investigation. The PBMC for this investigation were isolated from the blood samples collected at necropsy of the animals which varied between week 18 and 89 post-challenge, depending on the euthanization performed due to AIDS-related symptoms or end of the study. Plasma SIV RNA levels were determined by real-time RT-PCR analysis at the NIH core facility, as described previously [14]. The threshold sensitivity of the real-time RT-PCR assay is <100 RNA copy equivalents/ml of plasma with an inter-assay variation <25% (coefficient of variation). The viral loads in these animals ranged from <100 to 26×10^6^ copies/ml. Based on the plasma viral loads the monkeys were stratified arbitrarily in to three groups: long-term non-progressors (LTNP; n = 10) with low to undetectable viral loads (<1.0×10^2^ to 5.1×10^2^ copies/ml), chronically infected but clinically healthy (Chronic; n = 5) with moderate vial loads (0.1×10^4^ to 6.1×10^4^copies/ml), and highly viremic (Viremic; n = 5) exhibiting high viral loads (0.18×10^6^ to 26×10^6^copies/ml) and with AIDS-defining complications (including: weight loss, retroviral pneumonia, pleural fibrosis, type II pneumocyte hyperplasis, diarrhea, wasting, generalized encephalitis with malacia and gliosis multifocally in the brain).

### Cells stimulation

Cryopreserved PBMC samples were removed from liquid nitrogen and were rapidly thawed in a 37°C water bath, gently mixed, washed with RPMI-1640 (HyClone laboratories, Logan, UT) to remove the freezing medium and re-suspended in complete media (CM; RPMI-1640 supplement with 10% heat-inactivated FCS (HyClone laboratories), 2 mM L-glutamine (Sigma-Aldrich), 100 U/ml penicillin/streptomycin (Invitrogen), for culturing in 6-well tissue culture plates overnight at 37°C in a humidified 5% CO_2_ atmosphere. The next morning the viable cell counts were determined by the trypan-blue dye exclusion and adjusted to 10×10^6^/ml with CM. Cells were stimulated for intracellular cytokine staining as following: Aliquots of 0.1 ml cells suspensions (1×10^6^ cells) were placed in 96 wells tissue culture plate (BD Biosciences, Franklin lake, NJ) and cultured with phorbol 12-myristate 13-acetate (PMA) at 50 ng/ml and Ionomycin (I) at 500 ng/ml (both from Sigma-Aldrich, St. Louis, MO), in a total volume of 0.2 ml. An additional aliquot of the cells suspension without PMA+I served as negative control. The cells were cultured for 1.5 h before adding brefedin A (Sigma-Aldrich, St. Louis, MO) at 10 µg/ml (to prevent cytokine secretion from the cells), for an additional 4.5 h, to a total stimulation for 6 h. Subsequently, the cells were harvested by washing in cold (4°C) flow wash buffer (Dulbecco's PBS (DPBS, Ca2^+^/Mg2^+^-free; Life technologies, Rockville, MD, with 2% heat-inactivated FCS, HyClone laboratories) and then processed for staining various fluorescence labeled antibodies. As reported earlier, brefeldin A treatment not only is useful to retain cytokines within the cell cytoplasm, but also to sustain expression of any induced cells surface molecules [15]. Indeed, in this study we observed that stimulation of PBMC for a total of 6 hours (1.5 h with medium or PMA+I and the last 4.5 h in the presence of brefeldin A), caused no changes in the surface phenotype of the different cells subsets analyzed (data not shown).

### Antibodies

The fluorescence-labeled monoclonal antibodies CD3 PE-Cy7 (SP34-2), CD8 Alexa700 (RPA-T8), CD28 PerCP-cy5.5 (L293), CD95 (DX2), IFN-γ FITC (B27), IL-2 PE (MQ1-17H12) and IgG1 and IgG2 isotype matched controls were obtain from BD Biosciences (San Jose, CA). The monoclonal antibody CD4 Pacific blue (OKT4) was obtained from eBiosciences (San Diego, CA). The live-dead fixable dead cells stain kit was obtained from Invitrogen (Carlsbad, CA).

### Immunofluorescence staining and flow cytometry analysis

The intracellular cytokine staining assay was performed as described previously [16]. Briefly, stimulated cells were first stained with live/dead fixable aqua fluorescent reactive dye at 4°C in the dark for 30 minutes and washed once with cold flow wash buffer. Then, the appropriately titrated antibodies to cell surface were added to the cells and incubated for 30 minutes at 4°C in the dark. Both compensation controls and fluorescence minus one (FMO) controls were utilized. Surface stained cells were then washed with cold flow wash buffer and fixed in fixation solution (BD Biosciences; San Jose, CA) for at least 10 minutes before performing intracellular cytokine staining. Fixation/permabilization solution was used for intracellular cytokine staining following manufacturer's instructions (BD Biosciences; San Jose, CA). Appropriately titrated FITC-labeled anti- IFN-γ (B27) and PE-labeled anti-IL-2 (MQ1-17H12) were added to the permeabilized cells for 60 minutes at 4°C in the dark, followed by washing twice with permeabilization solution. After the final wash, the cells were re-suspended in 1% paraformaldehyde in DPBS and subjected to Flow cytometry analysis within 24 hours. The stained cells were acquired on a LSR II flow cytometer (BD Biosciences; San Jose, CA) or Cyan ADP (Dako, Carpinteria, CA), and FACS data were analyzed by using FlowJo software (Tree Star, Ashland, OR). In order to perform uniform analyses across the three groups of animals at different stages of SHIV infection, as defined by viral loads, we normalized the percentages of total T cells and the different subsets to an arbitrary total of 10,000 input peripheral blood lymphocytes (PBL).

### Statistics

Data are represented as mean ± SEM. The significance of differences between groups was determined using the unpaired, two-sided Student's t test, and *P* values <0.05 were considered significant different.

## Results and Discussion

**Figure 1:**
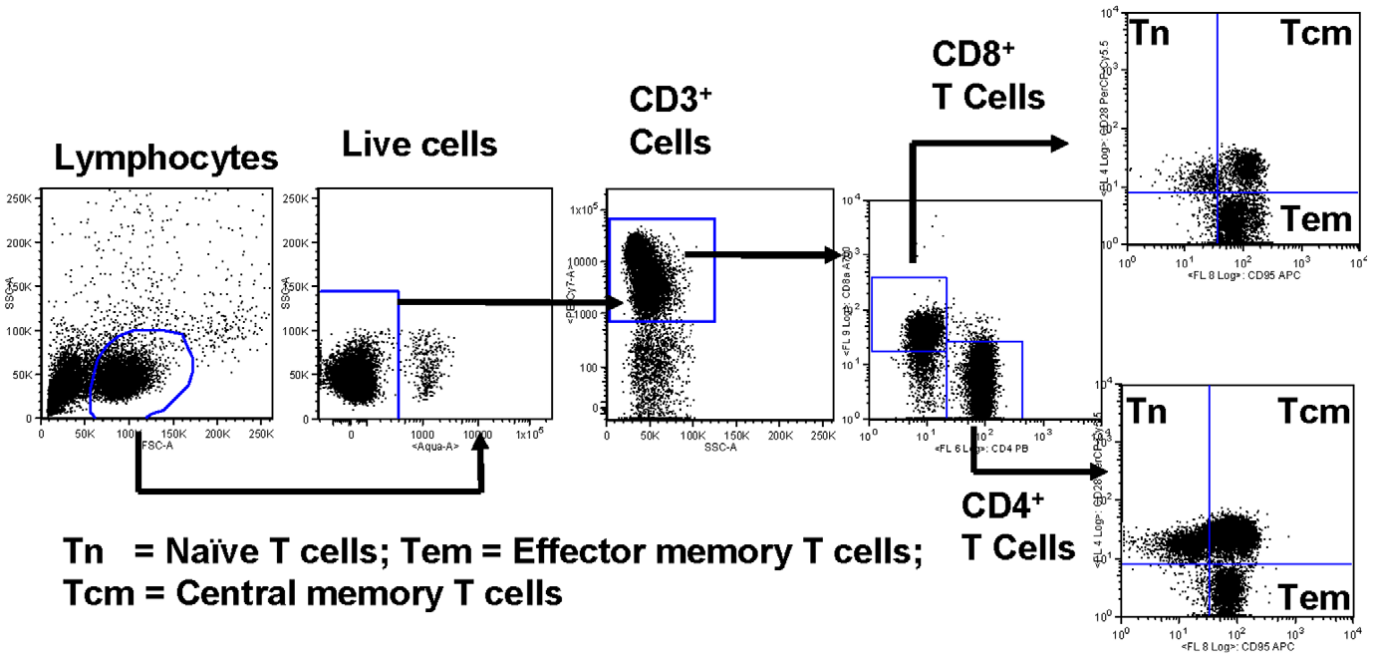
Gating scheme for the analyses of the different T cell subsets in the peripheral blood mononuclear cells (PBMC) from a representative animal. The lymphocytes were first gated based on forward scatter (FCS) versus side scatter (SSC), and then live lymphocytes were identified based on SSC and aqua-negative population. The T cells were then positively identified by CD3 expression followed by the detection of the CD4^+^ CD8^−^ (CD4^+^ T cells) and CD4^−^ CD8^+^ (CD8^+^ T cells) populations within the CD3^+^ T cells. On the basis of CD28 and CD95 expression, the CD4^+^ and CD8^+^ T cells were further differentiated into naïve (Tn CD28^+^ CD95^−^), central memory (Tcm CD28^+^ CD95^+^) and effector memory (Tem CD28^−^ CD95^+^) subsets.

We used multicolor flow cytometry analyses to investigate the functional features paralleling the circulating total and memory subsets of CD4^+^ and CD8^+^ T cells in SHIV-infected rhesus macaques exhibiting varying levels of plasma viral loads along with or without clinical symptoms. Peripheral blood mononuclear cells (PBMC) collected from a total of 20 rhesus macaques at the necropsy and preserved at liquid nitrogen were used for the multicolor flow cytometry analyses. These 20 animals were used in the past for vaccine studies [12], [13] and changes in viral loads and CD4^+^ T cell counts were recorded for approximately one year post-infection with SHIV (Fig. S1). Based on the viral loads and presence or absence of clinical symptoms at necropsy the animals were grouped as long-term non-progressors (LTNP, n = 10), chronically infected but healthy (Chronic, n = 5), and symptomatic with high viral loads (Viremic, n = 5), as described in the Methods section. The gating scheme used for the eight-color flow cytometry analyses of the different T cells subsets from a representative animal is shown in Fig. 1. The lymphocytes were first gated based on forward scatter (FSC) versus side scatter (SSC) with the dead cells excluded using the violet live/dead stain. The live T cells were then positively identified based on CD3 expression followed by detecting CD4^+^ CD8^−^ (CD4^+^ T cells) and CD4^−^ CD8^+^ (CD8^+^ T cells) populations. On the basis of CD28 and CD95 expression, the CD4^+^ and CD8^+^ T cells were further differentiated into naïve (Tn CD28^+^ CD95^−^), central memory (Tcm CD28^+^ CD95^+^) and effector memory (Tem CD28^−^ CD95^+^) subsets as described in the literature [15]. Next, the total and the different subsets of CD4^+^ and CD8^+^ T cells were assessed for functional capacity in terms of cytokine production (IFN-γ and/or IL-2) in response to stimulation with PMA and ionomycin (PMA+I). This analysis enabled identification of three functionally distinct populations of total as well as memory subsets of CD4^+^, CD8^+^ T cells, each producing IFN-γ and IL-2 alone or the two cytokines together (Fig. S2). Since these cytokines are important for sustaining memory (IL-2) and mediating effector function (IFN-γ), this analysis provides a snap shot of the quantity and quality of the inherent cell-mediated immunity in relation to viral load in the three different groups of monkeys studied.

### Numbers of total CD4^+^ and CD8^+^ T cells are differentially associated with viral loads

**Figure 2:**
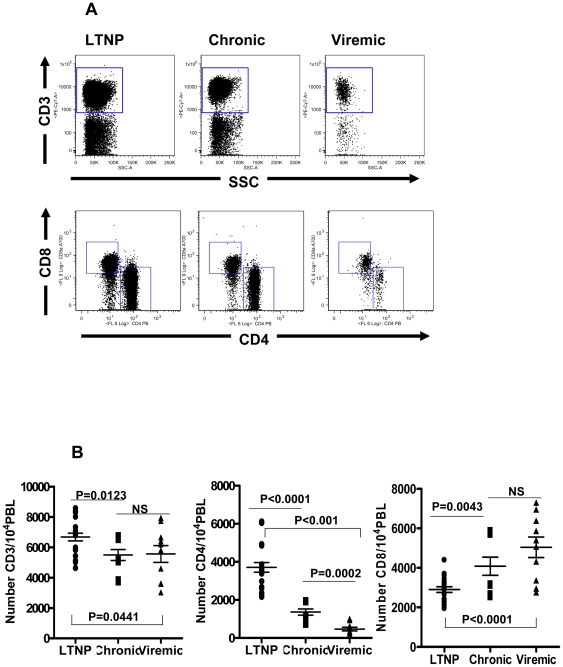
Flow cytometry analyses showing the distribution of total CD3^+^ T cells along with subsets CD4^+^ CD8^−^ (CD4^+^ T cells) and CD4^−^ CD8^+^ (CD8^+^ T cells). Panel A: data from a representative animal from each of the three groups of macaques with different viral loads: Long-term non-progressors (LTNP), chronic viremia (Chronic), and highly viremic (Viremic). Panel B: comparison of the average values for the numbers of total CD3^+^ T cells as well as CD4^+^ CD8^−^ and CD4^−^ CD8^+^ T cells subsets in all the animals studied in the LTNP, Chronic and Viremic groups of monkeys. The data are shown as the mean ± SEM. The Horizontal bars represent the group mean, error bars represent SEM. Statistical differences between groups are indicated by the *p* values.

The CD4^+^ T cells play an important role in maintaining effective immunity against viral pathogens, specifically by providing help to B cells for antiviral antibody production and to cytotoxic T lymphocytes (CTL) for eliminating virus-infected cells [17], [18]. We first examined the numbers of total CD3^+^ T cells as well as CD4^+^ and CD8^+^ T cells subsets in all the animals tested and representative data from one animal each in the three different groups (Fig. 2A). As described in the methods section, in order to perform uniform analyses across the three groups of animals as defined by viral loads, we normalized the data for the phenotypic and functional properties of the different subsets of T cells to an arbitrary total number of 10,000 input peripheral blood lymphocytes (PBL). Based on this criterion we observed that the number of total CD3^+^ T cells in the LTNP group (6676±254; Mean ± SEM) were significantly higher (p = 0.0123) compared to that in the Chronic group (5493±362) and also the Viremic group (5559±555; p = 0.0441), but no differences were noted between the Chronic and the Viremic groups (Fig. 2B). With regards to the CD4^+^ T cells, the mean number in the LTNP (3709±255) was significantly higher (p<0.0001) than that in the Chronic group (1362±163) and the Viremic group (464±96; p = 0.0002), as well as between the LTNP and the Viremic groups (p<0.0001). These results obtained using the arbitrarily selected total number of 10,000 PBL for the analyses are consistent with literature norms showing that both the Chronic and Viremic groups of monkeys with medium to higher viral loads exhibited significantly lower numbers of CD4^+^ T cells. The number of CD8^+^ T cells exhibited a reverse trend with the LTNP group (2901±145) significantly lower (p<0.0001) than the Viremic group (5042±519) and also the Chronic group (4081±459; p = 0.0043), but no differences were observed between the Chronic and the Viremic groups.

### Association of higher viral loads with decreased number of central memory and naïve subsets of CD4^+^ T cells

**Figure 3:**
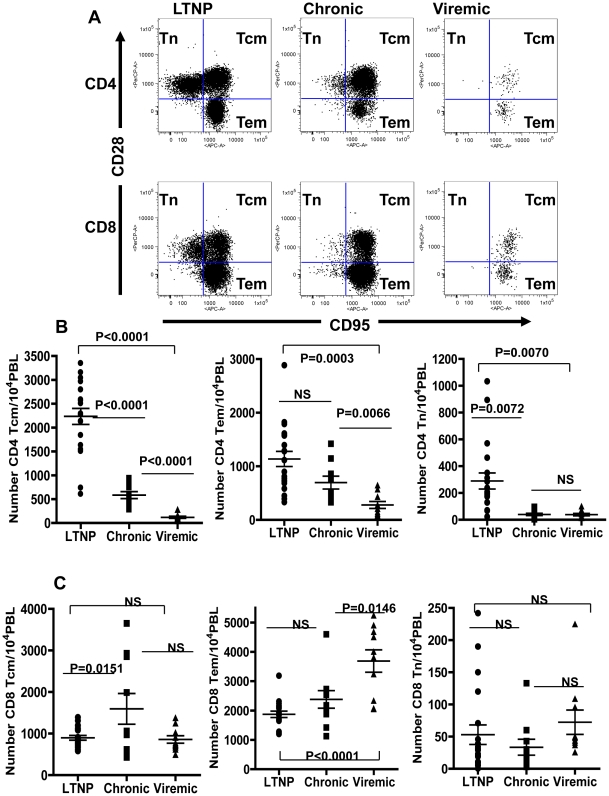
Flow cytometry analyses for the distribution of the memory subsets of the CD4^+^ and CD8^+^ T cells. **Panel A**: data from a representative animal from each of the three groups with different viral loads: Long-term non-progressors (LTNP), chronic viremia (Chronic), and highly viremic (Viremic). The memory cells were differentiated on the basis of CD28 and CD95 expression into naïve (Tn CD28^+^ CD95^−^), central memory (Tcm CD28^+^ CD95^+^) and effector memory (Tem CD28^−^ CD95^+^) subsets. **Panels B and C:** comparison of the average values for the numbers of the different memory subsets of CD4^+^ T cells (**B**) and CD8^+^ T cells (**C**) in all the animals tested in the LTNP, Chronic and Viremic groups of monkeys. The data are shown as the mean ± SEM. The Horizontal bars represent the group mean, error bars represent SEM. Statistical differences between groups are indicated by the *p* values.

It has been shown that both CD4^+^ and CD8 ^+^ T cells can be divided into two major memory subsets as central memory (Tcm) and effector memory (Tem) based on migration patterns and expression of phenotypic markers [19], [20] In particular, Tcm cells expressing CD28, CD95, CCR7 and CD62L home to lymph nodes, whereas Tem do not express CD28 or CCR7 and home to peripheral tissues that serve as effector sites [11], [21]. In case of nonhuman primates the Tcm and Tem subsets of CD4^+^ and CD8^+^ T cells can be identified on the basis of differential expression of CD28 and CD95 markers [15]. Using these markers we analyzed for central memory (CD28^+^ CD95^+^) and effector memory (CD28^−^ CD95^+^) subsets as well as naïve population (CD28^+^ CD95^−^) within the CD4^+^ CD8^−^ and CD4^−^ CD8^+^ T cells and representative FACS analysis patterns for one animal each for the three different groups are shown in Fig. 3A. Similar to reports in the literature [15] we did not observe changes in the levels of surface expression of CD28 and CD95 after treatment with PMI + I along with brefeldin A (data not shown). The Tem and Tcm subsets of CD4^+^ and CD8^+^ T cells were clearly evident even in the Viremic group of monkeys despite total numbers of CD3^+^ T cells being the lowest among the three groups. However, relative to that in the LTNP group monkeys, fewer numbers of naïve subsets of T cells were observed in the Chronic group animals and almost none were detectable in the Viremic group animals. In general, the Tem and Tcm subsets of both CD4^+^ and CD8^+^ T cells were clearly detectable in all three groups of animals (Fig. 3B and 3C, respectively). Comparative analyses of the different subsets of CD4^+^ T cells (Fig. 3B) revealed that the number of CD4^+^ Tcm in the LTNP group (2235±168, Mean ± SEM) was significantly higher (p<0.0001) compared to that in the Chronic group (585±74), which was in turn significantly higher (P<0.0001) than that in the Viremic group (118±22). There was also significant difference for the Tcm number between animals in the LTNP and the Viremic groups (p<0.0001). The number of naïve CD4^+^ T cells (CD4 Tn) in the animals in both the Viremic group (39±9) and the Chronic group (40±9) were significantly lower (p = 0.0072) when compared to that in the LTNP group (289±60) but no differences were observed for this population between the Chronic and the Viremic groups. These observations describing the significant loss of naïve CD4^+^ T cells (CD4 Tn) in the animals in both the Viremic group and the Chronic group, relative to that in the LTNP group, are in agreement with similar results reported in the literature [22]. There was no significant difference observed in the number of CD4 Tem (CD28^−^ CD95^+^) cells between the LTNP (1137±140) and the Chronic groups (696±119), but the numbers in the Viremic group (279±65) were significantly lower compared to that in the LTNP and the Chronic groups (p = 0.0066 and 0.0003, respectively). In contrast to the CD4^+^ T cells subsets, we observed no differences in the frequencies for the numbers of Tcm and Tn populations of CD8^+^ T cells between the three different groups (Fig. 3C), and with respect to the CD8^+^ Tem cells, significantly higher number were observed in the Viremic group relative to that in the LTNP animals (3688±380 versus 1873±109), but no differences were noted between the LTNP and the Chronic groups (1873±109 versus 2381±299). Thus, both the total and the Tcm subset of CD4^+^ T cells were significantly lower, in the Chronic group compared to those in the LTNP group, while no such differences were observed for the CD8^+^ T cells subsets. Our results are in line with observations reported in the literature that showed loss of CD4 Tcm subset as an important underlying mechanism for the deficiency to replenish gradually declining Tem subsets of both CD4^+^ and CD8^+^ T cells in animals with chronic SIV infection [23].

### Differential association of the numbers of IFN-γ and IL-2 producing total and memory subsets of CD4^+^ and CD8^+^ T cells with viral loads

**Figure 4:**
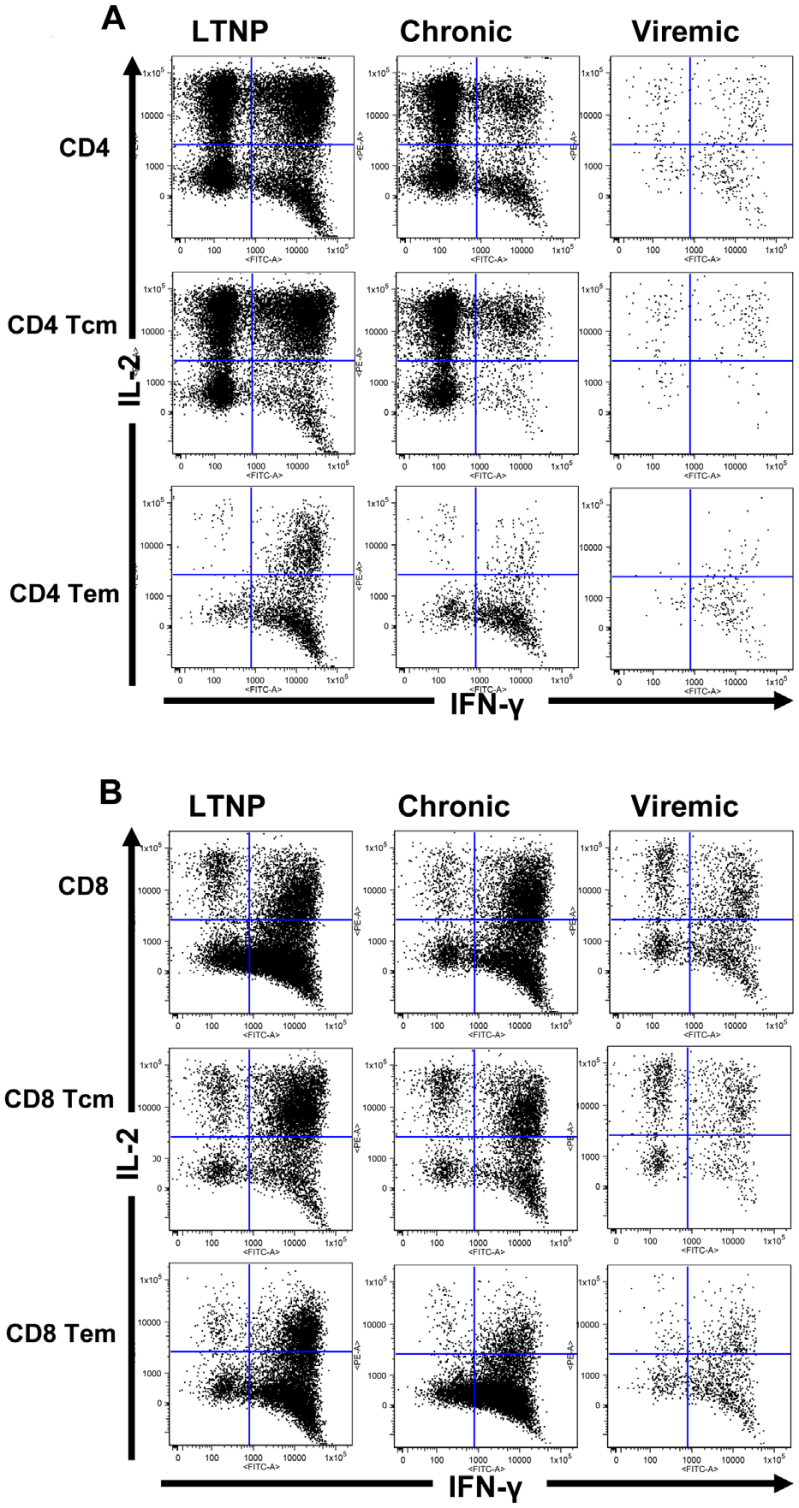
Flow cytometry analyses for the patterns of IFN-γ and/or IL-2 production profile of total as well as the Tcm and Tem memory subsets of CD4^+^ T cells (A) and CD8^+^ T cells (B) in a representative animal from each of the three groups with different viral loads: Long-term non-progressors (LTNP), chronic viremia (Chronic), and highly viremic (Viremic).

It is believed that different subsets of CD4^+^ and CD8^+^ T cells play different functional roles with respect to antiviral immunity [6]. To test the functional characteristics of total CD4^+^ and CD8^+^ T cells as well as their memory subsets in the three groups of monkeys with different viral loads, we determined production of IL-2 and/or IFN-γ in response to in vitro stimulation with PMA+I by cytokine flow cytometry analysis, a technique that allows for precise quantification and phenotypic characterization of cytokine producing T cells [24], [25]. We observed substantial frequencies for the numbers of IFN-γ producing cells in the total as well as memory subsets of both CD4^+^ and CD8^+^ T cells, and these responses were restricted almost entirely to the population defined by memory phenotype as shown for a representative animal from each of the three groups studied (Figs. 4A and 4B). In general, we observed CD4^+^ as well as CD8^+^ T cells producing both IFN-γ and IL-2 after PMA+I stimulation in all three groups exhibiting different viral load levels, with the Tcm predominately producing IL-2 and the Tem producing IFN-γ. This is consistent with the effector function of Tem subset and the precursor function of Tcm subset to maintain the homeostasis of Tem during viral infections.

**Figure 5:**
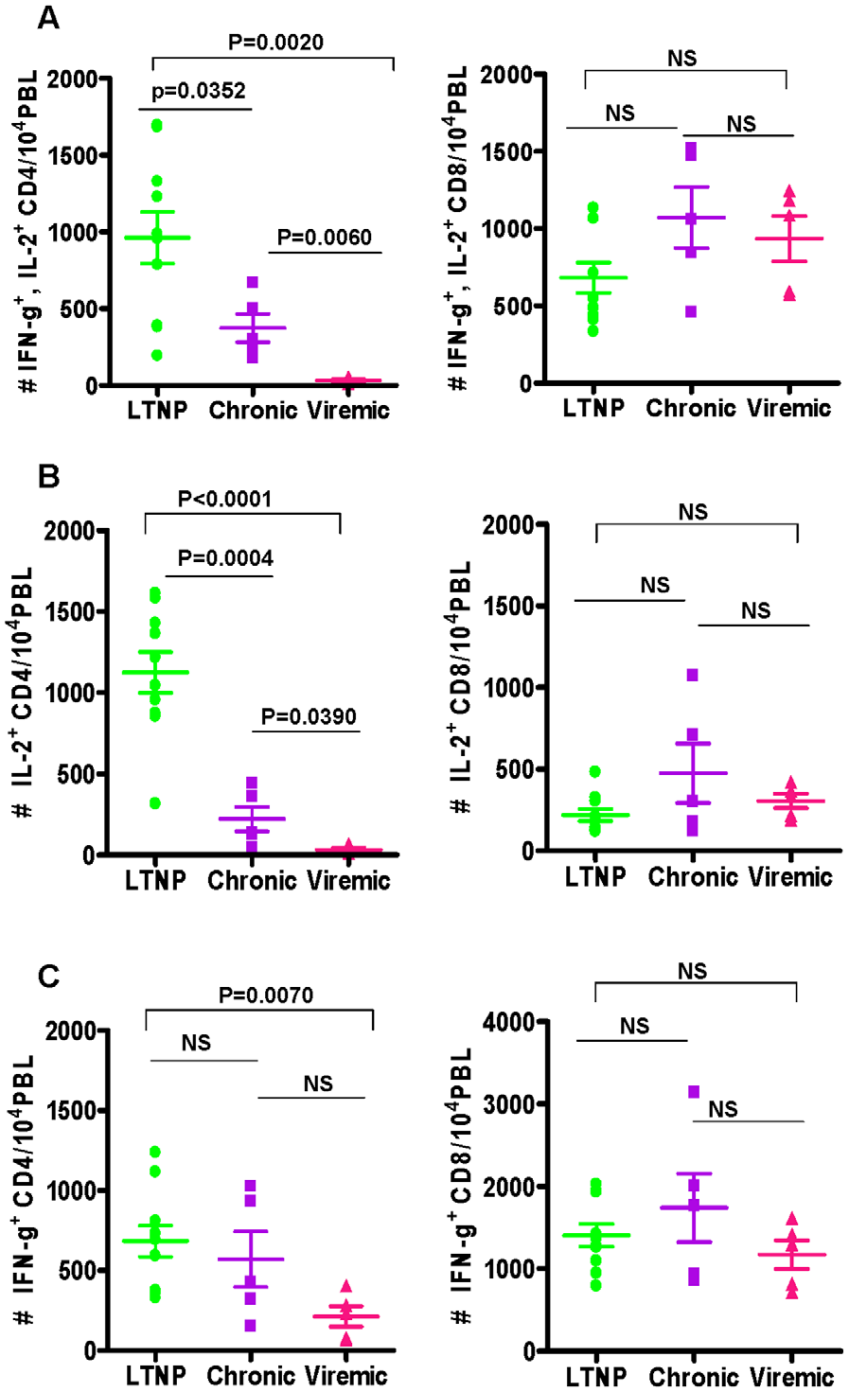
Comparison of the average values for the numbers of cytokine producing total CD4^+^ and CD8^+^ T cells in LTNP, Chronic and Viremic groups of animals: IFN-γ and IL-2 (A), IL-2 (B), and IFN-γ (C). The data are shown as the mean ± SEM. The Horizontal bars represent the group mean, error bars represent SEM. Statistical differences between groups are indicated by the *p* values.

A summary of the total CD4^+^ and CD8^+^ T cells producing IFN-γ and/or IL-2 in response to in vitro stimulation with PMA+I in all the tested animals is shown in Figs. 5A through 5C. The average number of total CD4^+^ T cells producing both the cytokines, as shown in Fig. 5A, is significantly higher in the LTNP group (963±168) compared to that in the Chronic group (375±92, p = 0.0352) and also the Viremic group (32±9, P = 0.006). However, unlike the total CD4^+^ T cells, the mean numbers of total CD8^+^ T cells producing both IFN-γ and IL-2 in the three different groups of animals were not significantly different from each other. With respect to the number of total CD4^+^ T cells producing a single cytokine in response to stimulation with PMA+I the average frequencies of IL-2 producing CD4^+^ T cells, as shown in Fig. 5B, animals in the LTNP group showed significantly higher numbers (1125±126) compared to those in the Chronic group (222±76) and the Viremic group (33±11). Also, the chronic group animals showed significantly higher numbers of IL-2 producing CD4^+^ T cells relative to that in the Viremic group. Unlike the CD4^+^ T cells, there is no significant difference in the three groups of monkeys for the frequencies of the numbers of total CD8^+^ T cells producing IL-2. We also compared the numbers of IFN-γ producing total CD4^+^ T cells in all the three groups of animals and observed that the LTNP group showed significantly higher numbers than that in the Viremic group but not the Chronic group and also no significant differences were observed between the Chronic and the Viremic group of animals (Fig. 5C). In contrast to the CD4^+^ T cells, there are no significant differences in the three groups of monkeys for the numbers of total CD8^+^ T cells producing IFN-γ. These data support the conclusion that the CD4^+^ T cells, but not the CD8^+^ T cells, selectively exhibit a significant impairment in the IL-2 producing capacity, but not IFN-γ in the Chronic group of monkeys with higher viral loads, relative that in the LTNP group of monkeys with low to undetectable plasma viral loads, even though both these groups of animals exhibited no signs of disease.

**Figure 6:**
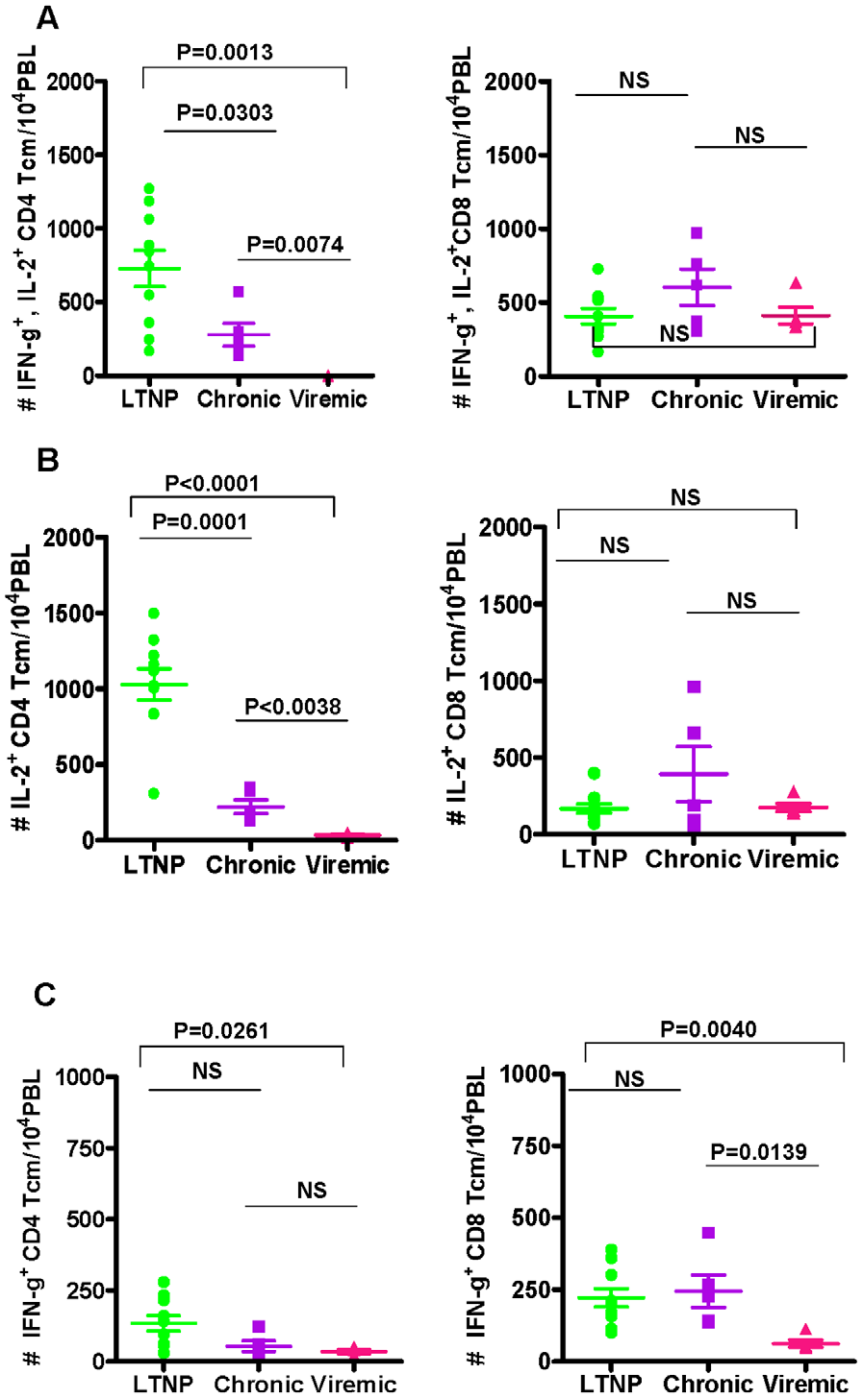
Comparison of the average values for the numbers of cytokine producing central memory CD4^+^ and CD8^+^ T cells (Tcm) in LTNP, Chronic and Viremic groups of animals: IFN-γ and IL-2 (A), IL-2 (B), and IFN-γ (C). The data are shown as the mean ± SEM. The Horizontal bars represent the group mean, error bars represent SEM. Statistical differences between groups are indicated by the *p* values.

**Figure 7:**
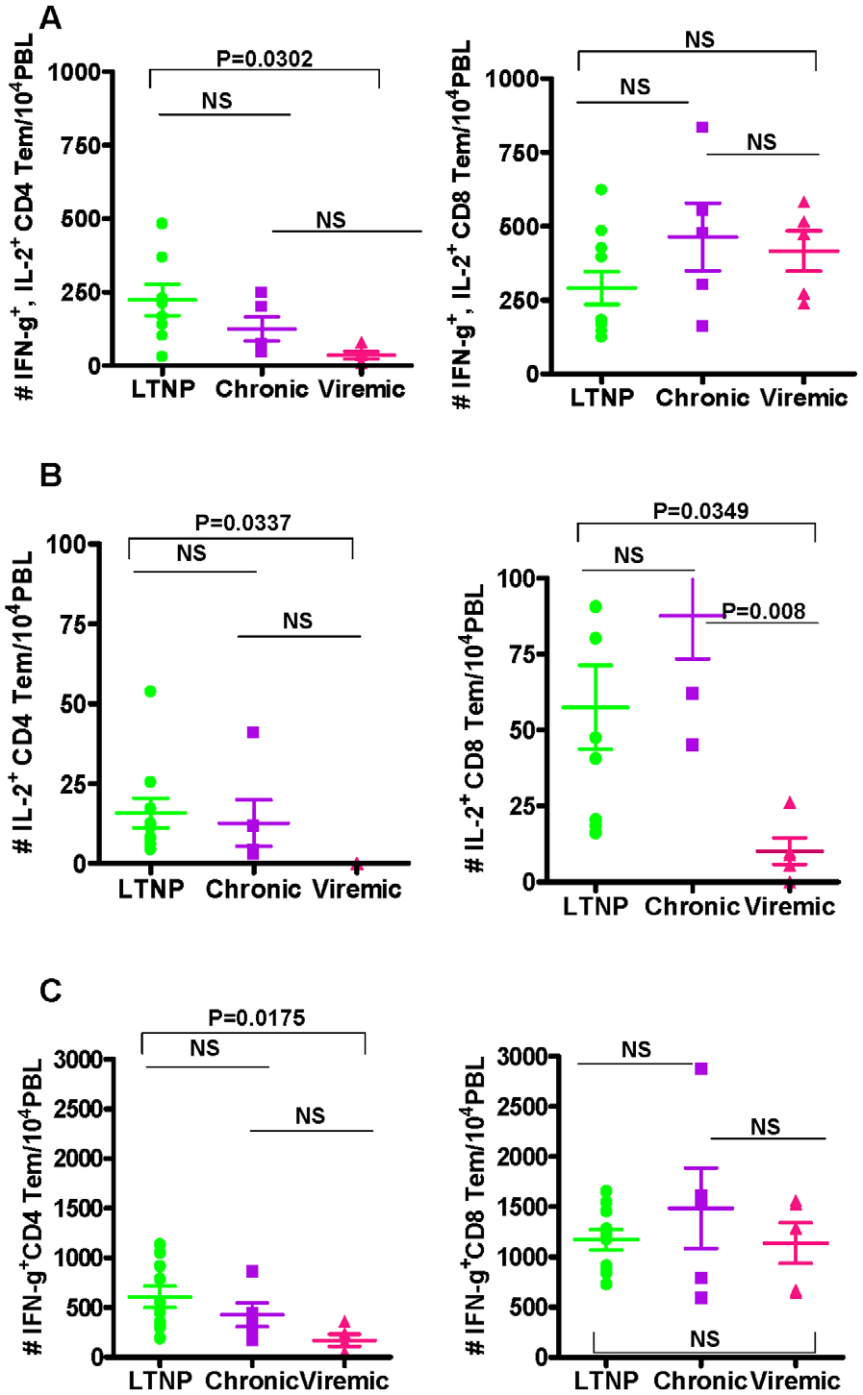
Comparison of the average values for the numbers of cytokine producing effector memory CD4^+^ and CD8^+^ T cells (Tem) in LTNP, Chronic and Viremic groups of animals: IFN-γ and IL-2 (A), IL-2 (B), and IFN-γ (C). The data are shown as the mean ± SEM. The Horizontal bars represent the group mean, error bars represent SEM. Statistical differences between groups are indicated by the *p* values.

Similar to the total CD4^+^ T cells, significantly higher numbers of duel cytokine producing CD4^+^ Tcm cells subsets were observed in LTNP group (728±124), followed by Chronic (277±77) and then the Viremic group (0.15±0.06) as shown in Fig. 6A. With respect to the CD8^+^ Tcm cells, we obtained results similar to that of the total CD8^+^ T cells, in that the average numbers of total CD8^+^ Tcm cells producing IFN-γ and IL-2 in the three groups are not different from each other (Figs. 6A). Analyses of Tcm subset of CD4^+^ T cells producing single cytokine revealed impairment for IL-2 production, but not IFN-γ production in the Chronic group of animals in terms of significantly lower numbers compared to those in the LTNP group while production of neither IL-2 nor IFN-γ was different for the Tcm subset of CD8^+^ T cells between these two groups (Figs. 6B–C). With respect to the CD4^+^ Tem cells subsets, only very few numbers of CD4^+^ Tem cells producing IL-2 were seen in the three different groups (Fig. 7B). We observed significant difference only between the LTNP group and the Viremic group, but not between the LTNP and the Chronic or the Viremic and the Chronic groups for IFN-γ and/or IL-2 production in response to PMA+I stimulation (Figs. 7A to C). For the CD8^+^ Tem population cells producing IL-2 and IFN-γ as well as IFN-γ alone were not significantly different between the three groups.

Studies describing functional analyses of different T cell subsets generally rely on IFN-γ production as a typical read out but accruing data suggest that IL-2 secreting CD4^+^ T cells represent a key component of an effective immune response [26], [27]. Since IL-2 is an essential growth factor for all subtypes of T cells, it is believed that the Tcm subset of CD4^+^ T cells by virtue of strong proliferation capacity combined with IL-2 producing ability are necessary for the continuous replenishment of Tem subsets that are the main effector population but depleted in acute as well as chronic phases of HIV/SIV infections. Since CD4^+^ T cells constitute the major IL-2 producing population that is important for maintaining functional integrity of other immune effectors including CD8^+^ T cells, a loss of IL-2 producing capacity of CD4^+^ T cells could produce a domino effect eventually for the overall immune deficiency. Therefore, our observation of the functional impairment of the total as well as Tcm subsets of CD4^+^ T cells, in terms of significant decline in IL-2 producing capacity, may be interpreted as an important event to predict future immunodeficiency in the chronically infected animals. Analyses similar to our present study in a bigger cohort of infected animals followed prospectively would be important to further substantiate the importance of selective deficiency in one or more cytokine producing memory subset of CD4 T cells as a preferred predictive marker of disease progression.

In summary, results from this investigation suggest functional impairment of CD4^+^ T cells, specifically the Tcm subset in the animals with chronic infection but no clinical symptoms of disease, as potentially an earliest form of immune dysfunction that may predict the ensuing disease progression in HIV infection. Importantly, this functional impairment of CD4 Tcm cells could be detected using relatively robust, but non-specific, immune stimulation reagents (PMA+I). This is specifically useful in situations of chronic infection where antigen-specific effector cells are in limiting numbers that preclude detailed analyses requiring stimulation with multiple viral antigens. Another important finding is that the impairment of CD4^+^ T cell function preceded the more commonly employed predictive marker, which is CD8^+^ T cell function, based on reports that showed disease progression to correlate with exhausted CD8^+^ T cells in HIV-infected humans as well as in animal models as determined by changes in the expression of phenotypic markers such as PD1 that indicate functional anergy [28]. It has therefore been generally believed that the exhausted phenotype would be indicative of functional impairment, while the current study actually analyzed for and detected functional impairment, specifically in the CD4^+^ T cells as a potentially earliest biomarker for disease progression. Our results are in line with observations from Louis Picker's group [23] who showed loss of CD4 Tcm population, and thereby their essential role as precursors for Tem cells, to underline the deficiency to replenish gradually declining Tem cells. Our data further adds to this conclusion by providing functional assessment of this important CD4 memory subset of T cells. After the major destruction of memory cells during the acute phase infection, a dramatic proliferation of Tcm occurs, probably as part of the homeostatic mechanisms to compensate for the loss [20]. Based on such observations it is suggested that the decline of CD4 Tcm subset may be the critical trigger that serves as the initiating event for the immune deficiency in general towards onset of AIDS in the macaque model, and by extrapolation to HIV infected humans [23]. Our results showing deficiency of total and central memory CD4 T cells in terms of inability to produce IL-2 alone or in combination with IFN-γ in response to non-specific stimulation further support a possible role for this functional phenotype that could be detected potentially by relatively simplistic routine analyses in clinical settings. Plasma viral load and CD4 cell counts are currently the major criteria for initiating treatment with highly active antiretroviral therapy. Some recent reports call attention to the doubt as when treatment should be initiated [5], [29], [30]. We believe that the simple analyses employing the robust non-specific stimulation used in this investigation, if conducted in prospective manner, could detect the potential triggering events of disease progression and therefore may aid in better designing of clinical management strategies for HIV-AIDS patients in terms of early intervention.

## Supporting Information

Figure S1 Numbers of peripheral blood CD4^+^ T cells and plasma viral loads in each of the three groups of animals for the present investigation that were used in the past for vaccine studies. Changes in the CD4^+^ T cell counts and viral loads (viral RNA copy equivalents/ml of plasma) for the LTNP group (A and B, respectively), Chronic group (C and D, respectively), and Viremic group (E and F, respectively) were recorded for approximately one year post-infection with SHIV. See Methods section for experimental details.
(TIF)

Figure S2 Typical results showing IFN-γ and/or IL-2 production profile of total as well as naïve and memory subsets of CD4^+^ T cells (**A**) and CD8^+^ T cells (**B**) in the PBMC of a representative animal in response to stimulation with PMA + Ionomycin or medium (negative control).
(TIF)
