# Supplementary figures and images for: Functional Impairment of Central Memory CD4 T Cells Is a Potential Early Prognostic Marker for Changing Viral Load in SHIV-Infected Rhesus Macaques
Source: PLoS One. 2011 May 13;6(5):e19607. doi: 10.1371/journal.pone.0019607 (PMC3094340; doi:10.1371/journal.pone.0019607)

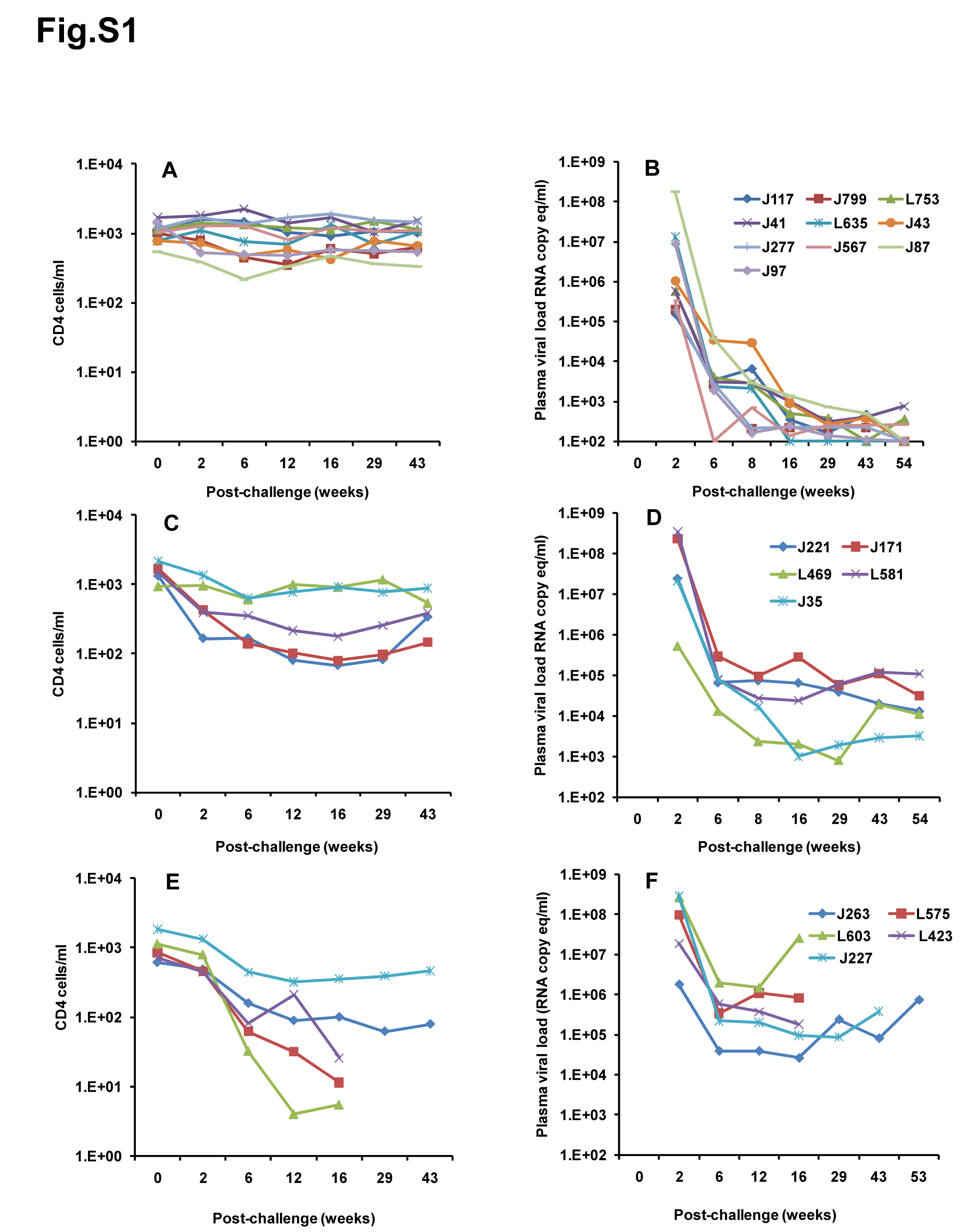

Supplement: Figure S1 — Numbers of peripheral blood CD4+ T cells and plasma viral loads in each of the three groups of animals for the present investigation that were used in the past for vaccine studies. Changes in the CD4+ T cell counts and viral loads (viral RNA copy equivalents/ml of plasma) for the LTNP group (A and B, respectively), Chronic group (C and D, respectively), and Viremic group (E and F, respectively) were recorded for approximately one year post-infection with SHIV. See Methods section for experimental details. (TIF) [file pone.0019607.s001.tif]

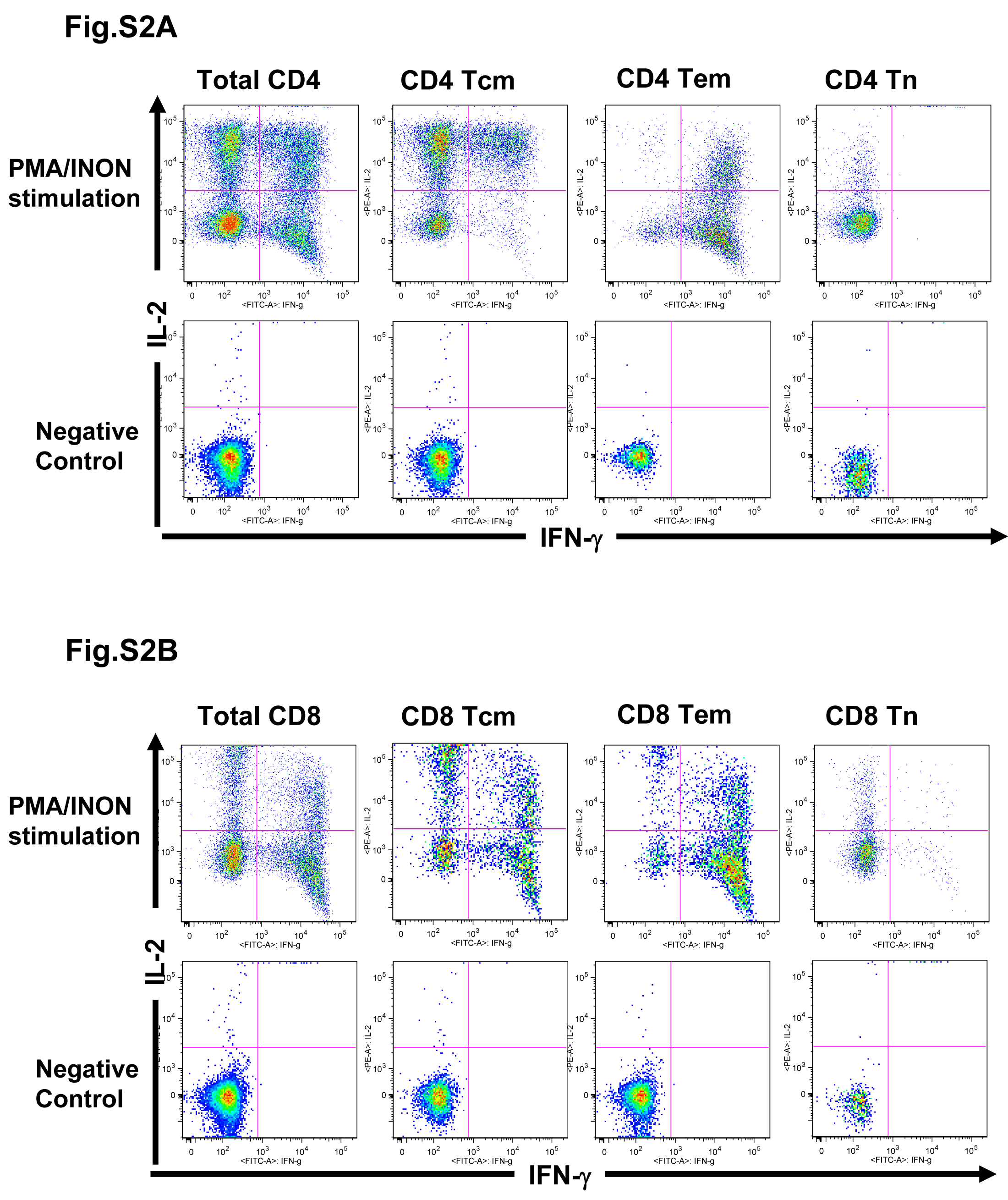

Supplement: Figure S2 — Typical results showing IFN-γ and/or IL-2 production profile of total as well as naïve and memory subsets of CD4+ T cells (A) and CD8+ T cells (B) in the PBMC of a representative animal in response to stimulation with PMA + Ionomycin or medium (negative control). (TIF) [file pone.0019607.s002.tif]
